# Shear fractures of the capitellum in children: a case report and narrative review

**DOI:** 10.3389/fsurg.2024.1407577

**Published:** 2024-07-04

**Authors:** Jean-Marc Pilotto, Silvia Valisena, Giacomo De Marco, Oscar Vazquez, Vincenzo De Rosa, Mario Mendoza Sagaon, Geraldo De Coulon, Romain Dayer, Dimitri Ceroni, Christina Steiger

**Affiliations:** ^1^ Service de Chirurgie Orthopédique et Traumatology de L’appareil Locomoteur, Hopitaux Universitaires de Genève, Genève, Switzerland; ^2^ Unité d’orthopédie et Traumatology Pédiatrique, Hopitaux Universitaires de Genève, Genève, Switzerland; ^3^ Servizio di Chirurgia e Ortopedia Pediatrica, Bellinzona, Switzerland

**Keywords:** capitellum, fracture, pediatric, osteosynthesis, shear fracture

## Abstract

The shear fractures of the capitellum are rare fractures in the pediatric population. Their diagnosis is challenging because of the high cartilaginous component of the growing elbow, requiring a high level of clinical suspicion especially in the case of small osteochondral or chondral fragments. The literature on capitellar shear fractures is mainly represented by case reports, which provides a patchy view of the topic. For this reason, we aimed to draw a narrative review presenting the available management strategies and their outcomes, and present two cases treated in our institution.

## 1 Introduction

The shear fractures of the capitellum are rare fractures both in children and adult patients. Due to the anatomical features of the elbow, their diagnosis can be often missed or delayed. For this reason, in the pediatric literature, these fractures have been grouped among the so-called TRASH injuries, an acronym that stands for “The Radiographic Appearance Seemed Harmless”, including also osteochondral fractures of the radial head and lateral condyle, incarcerated medial epicondylar and unossified medial condylar fractures, transphyseal separations of the distal humerus, and Monteggia lesions (1). The anatomy of the pediatric elbow is characterized by six ossification centers, merged in radio-transparent physes, which fuse during adolescence in a sequential order, beginning from the capitellum, trochlea and olecranon around the age of 14 (2). Consequently, any injury before the fusion of the ossification centres can cause chondral or osteochondral fragments that can be missed on radiographs, especially if small in size.

Capitellar shear fractures account for less than 1% of elbow fractures in both pediatric and adult patients (3–7). In children, they usually affect adolescents around the age of 14, just before the fusion of the ossification centre (8, 9). These fractures are generated by an axial load towards the humeral paddle with an elbow in extension or semiflexion, as in a fall on the outstretched arm (10–12). Depending on the flexion or extension of the elbow at injury, the fracture fragment can be anterior or posterior. These fractures can also be generated by a posterolateral elbow dislocation or subluxation, due to the impact of the radial head and coronoid against the capitellum (10–12). Even though in many cases they are isolated, the capitellar shear fractures can be associated with other injuries of the lateral column, such as radial head or neck fractures, lateral condyle fractures, lateral collateral ligament complex avulsion, but also medial condyle and olecranon fractures (13).

There are two main classifications of capitellar shear fractures in use for children and adults, the modified Bryan-Morrey and Dubberley (4, 14). The Bryan-Morrey classification distinguishes these injuries into three types, mainly depending on the fracture size of the fragment (4). Type 1, also called Hahn-Steinthal fracture, is an isolated fracture of the capitellum, characterized by a single fracture fragment including both the chondral and subchondral component of the capitellum, eventually extended to the lateral trochlear ridge. Type 2, the Kocher-Lorenz fracture, is a thin articular cartilage fragment of the capitellum. Type 3, also called Broberg-Morrey fracture, has the same extension as Type 1 but is comminuted. McKee added a fourth type to the Bryan-Morrey classification, that extends up to the trochlear groove (4).

Dubberley classified capitellar fractures into three types, differentiated by the extension in the trochlea, each one distinguished in type A and B, according to a more anterior or posterior involvement of the capitellum on the sagittal plane (14). In this classification, Type 1 is like a Hahn-Stenthal fracture, involving the lateral trochlear ridge, Type 2 describes a capitellar fragment that extends in the trochlear groove, and Type 3 is comminuted, with at least a capitellar and a trochlear fragment.

Due to the anatomy and the timing for the ossification of the pediatric elbow, the capitellar shear fracture diagnosis can be missed or delayed. In clinical practice, distinguishing these patterns of injuries requires not only a radiological diagnosis but also high clinical suspicion. The reason stands in the high cartilaginous component of the pediatric elbow, making impossible a direct radiographic diagnosis of small chondral fragments. Radiographs rather show a double contour sign, when the fracture involves the subchondral bone or just a fat pad sign in the case of a small chondral fragment (11). What leads clinicians to further investigate the injured elbow is the clinical appearance of a swollen and tender elbow, eventually accompanied by hematoma. The limitation of motion is not only related to pain but also to a mechanical blockage of the elbow amplitude related to the interposition of the fragment. In these cases, investigations are usually pursued with a computed tomography scan (CT) or magnetic resonance imaging (MRI) of the elbow, to characterize the fracture pattern and plan the surgery. The surgical approach and osteosynthesis or fragment excision are planned based on the imaging, leaving margins for the intraoperative adaptation of the strategy. Since there is no consensus on the best management, we aimed to describe two cases treated in our institution and provide a narrative review of available pediatric literature.

### 1.1 Case report

After approval by the local ethics committee, we retrospectively reviewed the charts of patients who accessed the Pediatric Emergency Service from January 2010 to December 2023 for traumas of the upper limbs. The age limit for admission to pediatric care in Switzerland is 16 years. Two patients with a capitellar shear fracture of the elbow were retrieved and are here described.

### 1.2 Case 1

**Figure 1:**
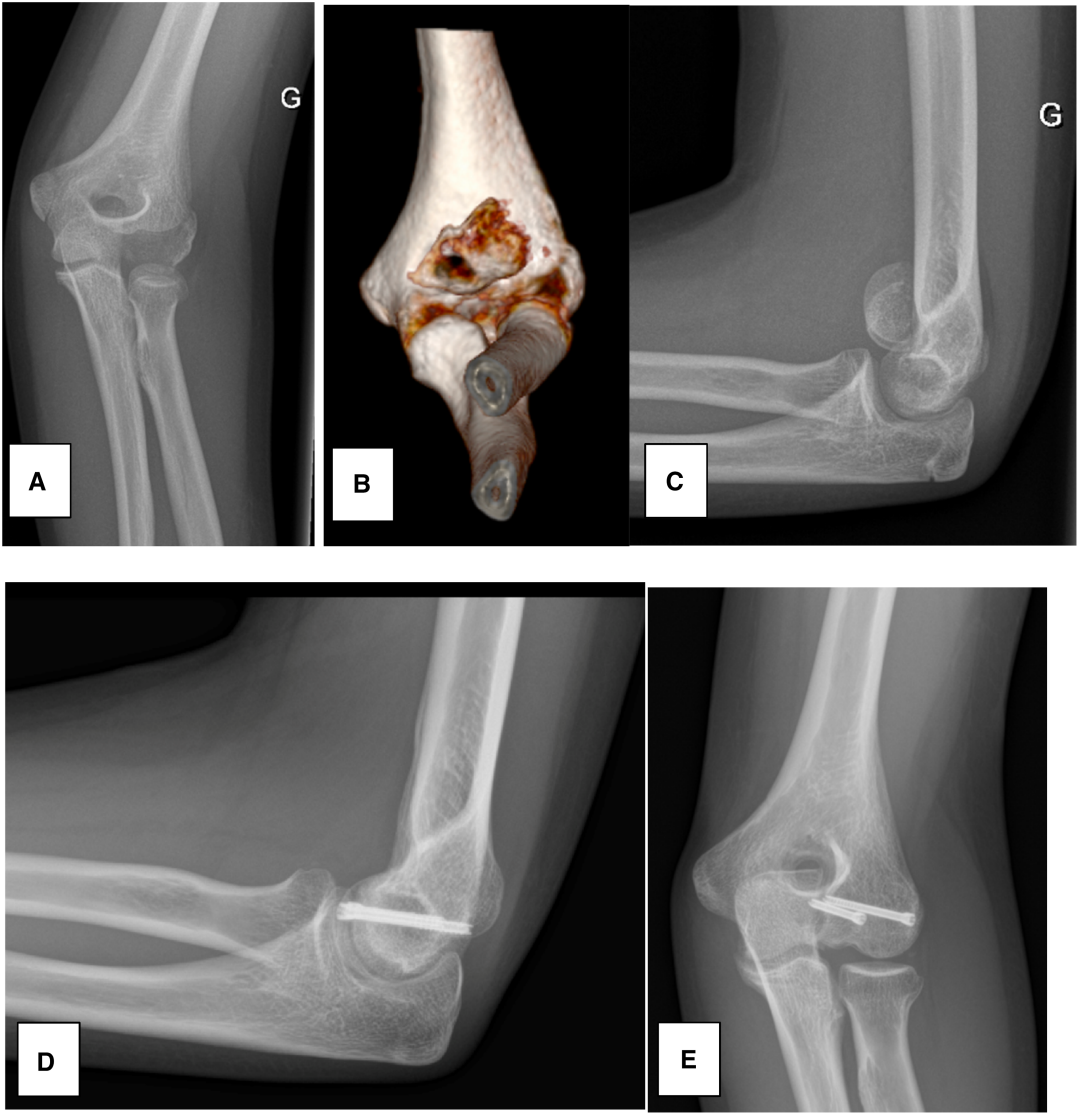
Case 1. (**A**) AP radiograph at admission. (**B**) 3D CT-scan showing the capitellar shear fracture, displaced towards proximal and medial. (**C**) Lateral radiograph at admission, showing the displaced capitellar shear fracture. (**D**,**E**) AP and lateral of the 1-year follow-up.

An 11-year-old female fell from the horse, with a trauma to her left upper limb and abdomen. The patient complained of pain in her left elbow and renal lodge. At the emergency department, the radiographs of the left elbow showed a double contour sign and a bone age of 12.5 years according to the Sauvegrain method (15). The elbow was then temporarily immobilized in a soft splint and a CT scan was scheduled. The blood and urinary test as well as an abdominal ultrasound allowed to exclude any renal or visceral injury. The CT scan of the elbow showed a shear fracture of the capitellum involving the lateral trochlear ridge, a Hahn-Steinthal fracture, with proximal and medial displacement. The surgery was scheduled for the following day. Through an anterolateral approach with distal anterior extension, through a deep plane between the brachioradialis and pronator teres, the fracture was exposed and prepared. The fracture was reduced and fixed by two K-wires, that guided the insertion of two headless cortical screws taking care to bury their anterior extremity. The intraoperative radioscopic check showed an adequate reduction and correct screw length, also confirmed by the postoperative radiographs. The elbow was immobilized in a brachioantebrachial (BAB) cast for 10 days. The postoperative follow-up lasted one year. The recovery occurred without complications, with a gradual improvement of the range of motion, requiring physiotherapy, and evidence of bone union at 2 postoperative months. At the final follow-up, the range of motion of the left elbow was identical to the right one (Figure 1).

### 1.3 Case 2

**Figure 2:**
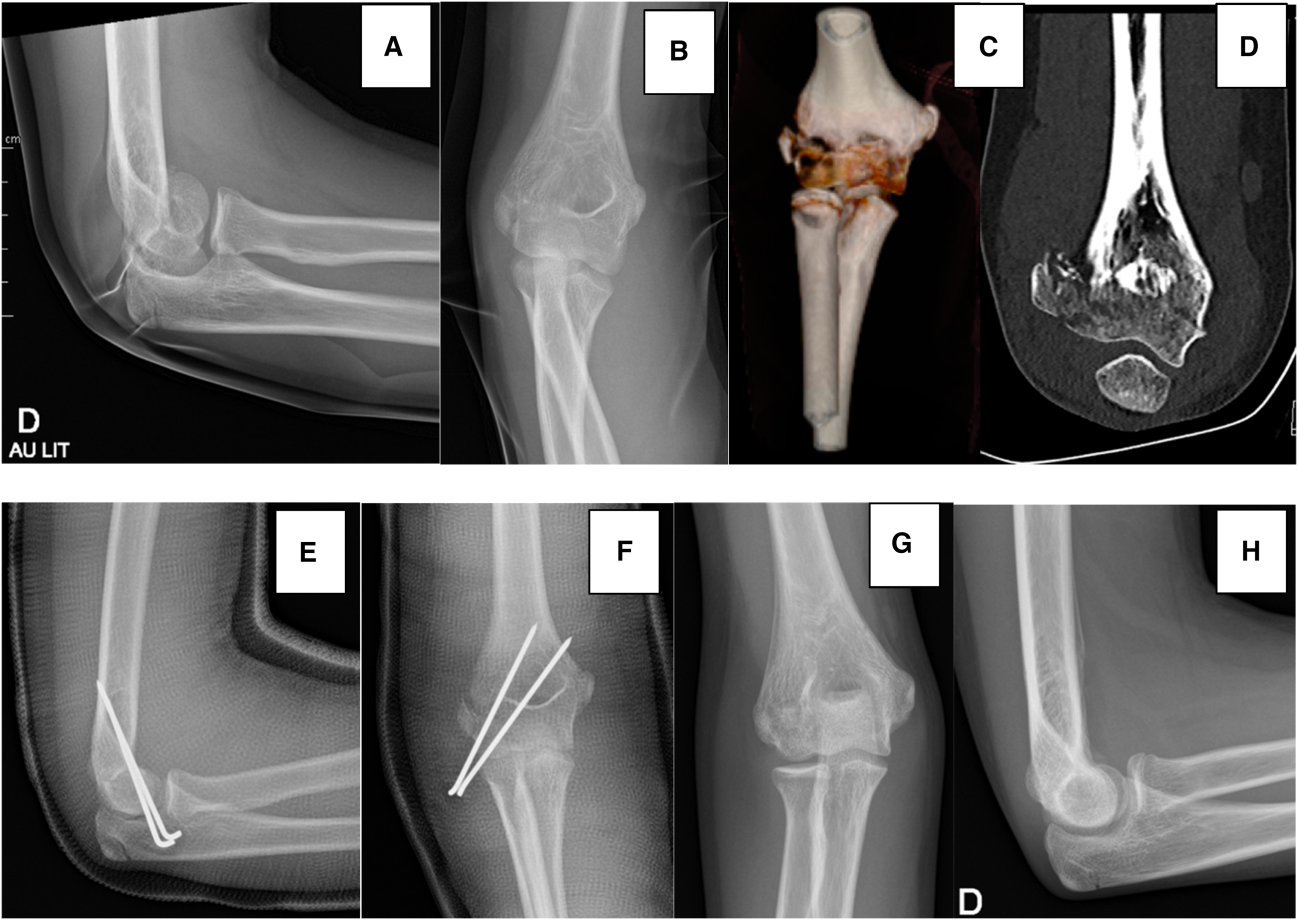
Case 2. (**A**) Lateral radiograph at admission, showing the double contour sign, (**B**) AP radiograph at admission. (**C**) 3D CT-scan showing the extension of the fracture towards the trochlear groove. (**D**) Coronal slice of the CT-scan showing the lateral displacement of the fragment. (**E**,**F**) Postoperative radiographs. (**G**,**H**) 2-month follow-up radiographs.

This is the case of an 11-year-old female who fell from monkey bars at school, with a trauma of her right upper limb. As she arrived at the emergency, she complained of pain in her right elbow, which appeared swollen and tender, and showed a reduced range of motion. The radiographs showed a double contour sign and a 12.5-year bone age according to the Sauvegrain method (15). The CT scan showed a McKee fracture extending up to the trochlear groove, with proximal and lateral displacement. The patient was hospitalized and surgery was scheduled for the following day. The fracture was approached by an anterolateral approach, reduced and fixed by two K-wires, whose extremities were kept subcutaneously at the closure. The elbow was immobilized in a BAB cast for 4 weeks when the K-wires were removed. At 10 postoperative weeks, the patient achieved a full range of motion and was allowed to gradually begin sportive activities. The radiographs showed the union of the fracture. The follow-up is still ongoing, with a planned consultation and 6 and 12 postoperative months (Figure 2).

## 2 Discussion

We carried out a narrative review of the pediatric literature for capitellar shear fractures (shown in Table 1). Most of the literature consists of case reports (5–8, 13, 16, 17, 20–23, 25–27, 29–31, 33, 38), followed by a smaller number of retrospective case series (9, 18, 19, 24, 28, 32, 34–37). The retrieved literature showed a bimodal distribution of these fractures, with a first peak in childhood (<10 years) (7, 17, 20, 31) and a second one, the most representative, in pre-adolescents and adolescents (5–9, 13, 16, 18, 19, 21–25, 27–30, 32–38). The most frequent associated injury was the ipsilateral elbow dislocation, followed by the involvement of the medial or lateral condyle, or an olecranon or radial head fracture (5, 16, 17, 21, 31, 37, 38).

**Table 1:** Narrative review for shear fractures of the capitellum in children.

Study	Sample size	Classification	Age	Associated injuries of the ipsilateral arm and elbow	Timing of diagnosis	Diagnostic imaging	Treatment	Postoperative complications	Follow-up duration (months)	Hardware removal (months)
Johansson and Rosman (5)	2	Bryan-Morrey classification: Type 1 and 2	12 and 13	Lateral condyle; radial neck	Acute	XR	ORIF with K-wire; excision via an open approach	Limitation of ROM (extension); radiocapitellar arthrosis	12–60	K-wires: 5 weeks
Agins and Marcus (6)	1	Articular cartilage sleeve fracture of the capitellum (n.c.)	11	Any	Acute	XR	ORIF (lateral approach) with K-wire	Limitation of ROM (extension)	6	K-wires: 8 weeks
Drvaric and Rooks (7)	1	Anterior sleeve fracture of the capitellum (n.c.)	8	Any	Acute	XR, arthrography	ORIF with Steinmann pins	Limitation of ROM (extension)	21	n.a.
Inoue and Horii (16)	1	Combined shear fracture of the trochlea and capitellum (n.c.)	11	Elbow dislocation	Acute	XR	ORIF (posterior approach) with 2 headless screws	Limitation of ROM (extension)	6	Any
Stricker et al. (17)	1	Salter Harris 4, including capitellum and trochlea	3	Ipsilateral proximal humeral fracture and elbow dislocation	Acute	XR	ORIF (posterior transolecranon approach) with K-wires	Any	36	K-wires: 4 weeks
Letts et al. (18)	7	Dubberley classification: Type 1 and 2	14.7 (mean)	Any	n.a.	XR	Conservative (2/7) ORIF with K-wires or screws	Limitation of ROM (extension) in 3 cases; Exostosis requiring operation (1 patient)	7.5 months to 3 years	n.a.
De Boeck and Pouliart (19)	6	Hahn-Steinthal	13.5 (mean)	Any	n.a.	XR	ORIF with screws	Limitation of ROM (flexion)	56	Any
Pradhan et al. (20)	1	Hahn-Steinthal	6	Any	Acute	XR	ORIF (lateral approach) with K-wires	Any	24	K-wires: n.a.
Bilić et al. (21)	1	Hahn-Steinthal	11	Ipsilateral elbow dislocation	Delayed (3 months)	XR	Conservative	Malunion requiring corrective osteotomy	24	Any
Sodl et al. (22)	1	Bryan-Morrey classification: Type 2	12	Any	Delayed (5 weeks)	XR, MRI	ORIF (Kocher approach) with 2 mattress sutures in “x” configuration	Any	14	Any
Cottalorda and Bourelle (13)	1	Kocher-Lorenz	12	Any	Delayed (3 years later)	XR, CT	Lateral approach and fragment excision	Any	96	Any
Suresh (23)	2	McKee	15 and 17	Any	Acute	XR, CT	ORIF (Kocher approach) with screws	Limitation of ROM (extension) in one patient	12	12 months
Ong and Mahadev (24)	9	Bryan-Morrey classification: Type 1	14 (mean)	Any	n.a.	XR	Conservative in two; ORIF (posterolateral approach) with cancellous screws and a K-wire in one patient	Keloid in one patient	7	4.7
Silva and Moazzaz (25)	1	Bryan-Morrey classification: Type 4	11	Any	Acute	XR, CT	ORIF (Kocher approach) with 2 headless screws	Any	6	Any
Cornelius et al. (26)	1	Salter Harris 3 fracture of the capitellum	9	Any	Acute	XR, MRI	ORIF (anterolateral approach) with a bioabsorbable pin	Any	12	Any
Gonçalves Pestana et al. (27)	1	Hahn-Steinthal	11	Any	Acute	XR	ORIF (posterolateral approach) with screws	Any	12	Any
Kurtulmus et al. (28)	8	McKee	15 (mean)	Any	n.a.	XR	ORIF (lateral approach) with screws	Trochlear irregularity (in one patient); keloid (in one patient)	26.4	Any
Dressler and de Paula (29)	1	McKee	16	Any	Acute	XR	ORIF (anterior approach) with headless screws	n.a.	n.a.	n.a.
Frank et al. (30)	3	Bryan-Morrey classification: Type 2	12–14 (range)	Any	Delayed (4–6 months)	XR, CT	Conservative	Chronic functional limitation; Radio-capitellar arthritis requiring surgery (radial head excision and joint capsular release)	4.5–12 (range)	n.a.
Fuad et al. (31)	1	McKee	9	Lateral collateral ligament avulsion	Acute	XR, CT	ORIF (Kaplan approach) with headless screws	n.a.	n.a.	n.a.
Murthy et al. (9)	37	Murthy classification: Type 1, 2, 3	11.8 (mean)	Any	n.a.	XR, CT or MRI	3 treatment types: Conservative (for non-displaced fractures) ORIF (Kocher approach) Arthroscopic or open fragment excision with or without microfracture	ORIF group: osteonecrosis and soft-tissue contracture, osteonecrosis with implant prominence, intra-articular loose fragments, or soft-tissue contracture alone	12.3 (mean)	One case of painful posterior prominence of a headless screw requiring removal
Onay et al. (32)	13	McKee	13.8 (mean)	Any	n.a.	XR, CT	ORIF (lateral or posterior transolecranon approach) with headless screws or K-wires	Limitation of ROM requiring hardware removal and arthrolysis in two patients; Arthritis in 3 patients; avascular necrosis of the capitellum in one patient	45	n.a.
Papamerkouriou et al. (33)	1	Bryan-Morrey classification: Type 4	14	Any	Acute	XR, CT	ORIF (Kocher approach) with headless screws and one percutaneous K-wire	Any	15	Percutaneous K-wire (at 3 weeks)
Nagda et al. (8)	2	Kocher-Lorenz	12 and 15	Any	Delayed (4–5 months from injury)	XR, CT, MRI	Kaplan approach, fragment excision, radial head excision and arthrolysis in one case, chondral fragment excision, microfracture on the capitellum for the other	Limitation of ROM (extension)	12	Any
Ju et al. (34)	15	Coronal fractures of the capitellum (n.c.)	13.1	n.a.	Acute	XR, CT	ORIF (anterior approach) with headless screws	Limitation of ROM	19.2	Any
Wiktor and Tomaszewski (35)	6	Bryan-Morrey classification Type 1 Type 4	13.2 (mean)	Any	n.a.	XR, CT	ORIF with absorbable screws	Limitation of ROM (extension or supination) in 2/6	12	Any
Kraus et al. (36)	51	n.a.	12.9 (mean)	n.a.	Acute (up to two weeks after trauma)	XR, CT or MRI	ORIF (lateral, bilateral, posterior, anterior approach) with screws or plates or K-wires or absorbable implants or anchors; one case of fragment excision	Limitation of ROM; persistent pain; revision of the osteosynthesis (4 patients); removal of a free joint body (1 patient); an osteonecrosis (1 patient); cartilage defect (1 patient)	9.9	Any
Simanovski et al. (37)	12	Dubberley classification: Type 1–3	13.2 (mean)	6/12 patients: -2 radial head -1 lateral condyle -1 medial condyle -1 dislocation -1 olecranon fracture	Acute (in one case), Delayed to 10 weeks in the others	XR, CT	ORIF (Kocher approach or posterior approach with olecranon osteotomy) with headless screws or an absorbable pin	Limitation of ROM (extension) in 7/12; Radiocapitellar arthrosis in 3/12	22	Any
Cao et al. (38)	1	Coronal shear fracture of the distal humerus (n.c.)	14	Radial collateral ligament injury, avulsion fracture of the right olecranon	Acute	XR, CT	ORIF (anterolateral approach) with custom-made plate	Any	14	14

Only studies on pediatric capitellar shear fractures were included in this narrative review.

Age, in years; CT, computed tomography scan; MRI, magnetic resonance imaging; n.a., not available/not applicable; n.c., not classified by the authors of the original paper; ORIF, open reduction and internal fixation; XR, radiographs.

The radiographs are the baseline exam for diagnosis, accompanied by a clinical suspicion. Before the advent of CT and MRI, arthrography was perioperatively used in selected cases or oblique radiographs were carried out to better visualize the fragments (7, 20). The use of CT and MRI has improved the diagnosis of capitellar shear fractures, allowing us to visualize them early and plan the treatment. According to the time elapsed between the accident and the management, it is possible to distinguish between fractures that are diagnosed early in the acute post-traumatic period or whose diagnosis is delayed. The availability of CT and MRI, within the first weeks from the injury, allowed setting a threshold of two weeks between the acute and delayed diagnosis (36). In fact, in most of the studies on the delayed finding of capitellar shear fractures, the diagnosis was stated between five post-traumatic weeks up to three years, with most of the cases between 3 and 6 months from the accident (8, 13, 21, 22, 30, 37).

As far as the classification is concerned, the most used one was the Bryan-Morrey, followed by the Dubberley. A few studies published in the 80ies and 90ies were rather descriptive in the definition of the fracture or used the Salter-Harris classification (6, 7, 16, 17). An orthopedic surgery team from Harvard provided their classification, based on the sagittal appearance of the capitellar shear fractures (9). By reversing the perspective provided by Dubberley, they proposed a classification distinguishing between anterior (Type 1) and posterior (Type 2) osteochondral fractures of the capitellum and chondral fractures (Type 3) (9). The perspectives offered by the mentioned classifications open the discussion for surgical planning. One of the most important elements for the planification of the surgical approach is a coronal extension of the fracture and any associated injury requiring surgical management (13, 18). The available literature shows that the most used approach is the lateral, especially through the Kocher interval between the anconeus and extensor carpi ulnaris (also called the posterolateral approach) (39). This allows a good exposure of both posterior and anterior fractures of the capitellum. For most anterior fractures, the options are the lateral approach through the Kaplan interval, between extensor carpi radialis brevis and extensor digitorum communis, but in rare cases also the anterior and anterolateral approach, especially for large fragments anteriorly displaced and with medial extension or in case of associated injuries to the lateral column (13, 18). To a lesser extent, also bilateral approaches or the posterior (eventually transolecranon) approach have been used (16, 17, 32, 36, 37).

The most used devices for osteosynthesis are screws and Kirschner wires. The latter were usually percutaneously fixed, allowing the removal between three to eight postoperative weeks (5, 6, 17, 20, 33). The headless screws, put on the sagittal plane of the fractures, have taken the place of the cancellous screws, not requiring another surgery for hardware removal, and being well tolerated in most of the cases. Some authors have also used osteosuture (22) or bioabsorbable pins (26, 35, 37). In the case of small fragments generating a mechanical blockage in the joint, whose size does not allow any fixation, these fractures are accessible for excision by an open or even arthroscopic approach (9). Finally, capitellar shear fractures can also be accessible to the conservative treatment for non-displaced fractures (9, 18, 21, 24, 30). In brief, the surgical indication lies in a symptomatic displaced fracture of the capitellum, where the most relevant determinant for surgical management is a mechanical blockage to the elbow range of motion.

When considering the timing of diagnosis, most of the cases with a prompt diagnosis were managed with open reduction and internal fixation. This was probably related to the fact that the fractures characterized by a remarkable displacement were promptly diagnosed on conventional radiographs. The fractures whose diagnosis was delayed were susceptible to all treatment strategies, from a conservative attitude when the clinical status allowed, rather than fragment excision or fixation (8, 13, 21, 22, 30).

In many cases, the treatment allows good and uneventful clinical and radiological outcomes. Among the postoperative complications, we can distinguish between the clinical manifestations with limitation of function, especially extension, persistent pain and even blockage. On one hand, the limitations to the elbow amplitudes can be related to soft tissue contractures, eligible for arthrolysis when the functional arc of motion is affected. On the other, pain and blockage can be the epiphenomenon of radiocapitellar osteoarthritis, capitellar necrosis and, in rare cases, loose fragments or cartilage defects, requiring surgical management (5, 9, 21, 30, 32, 36, 37). When distinguishing between the early and delayed diagnosis, the available literature showed that there was not a propensity for radiocapitellar osteoarthritis and capitellar necrosis in delayed cases, but these seemed to be equally distributed. One case of malunion requiring a corrective osteotomy was also described in the literature (21).

**Figure 3:**
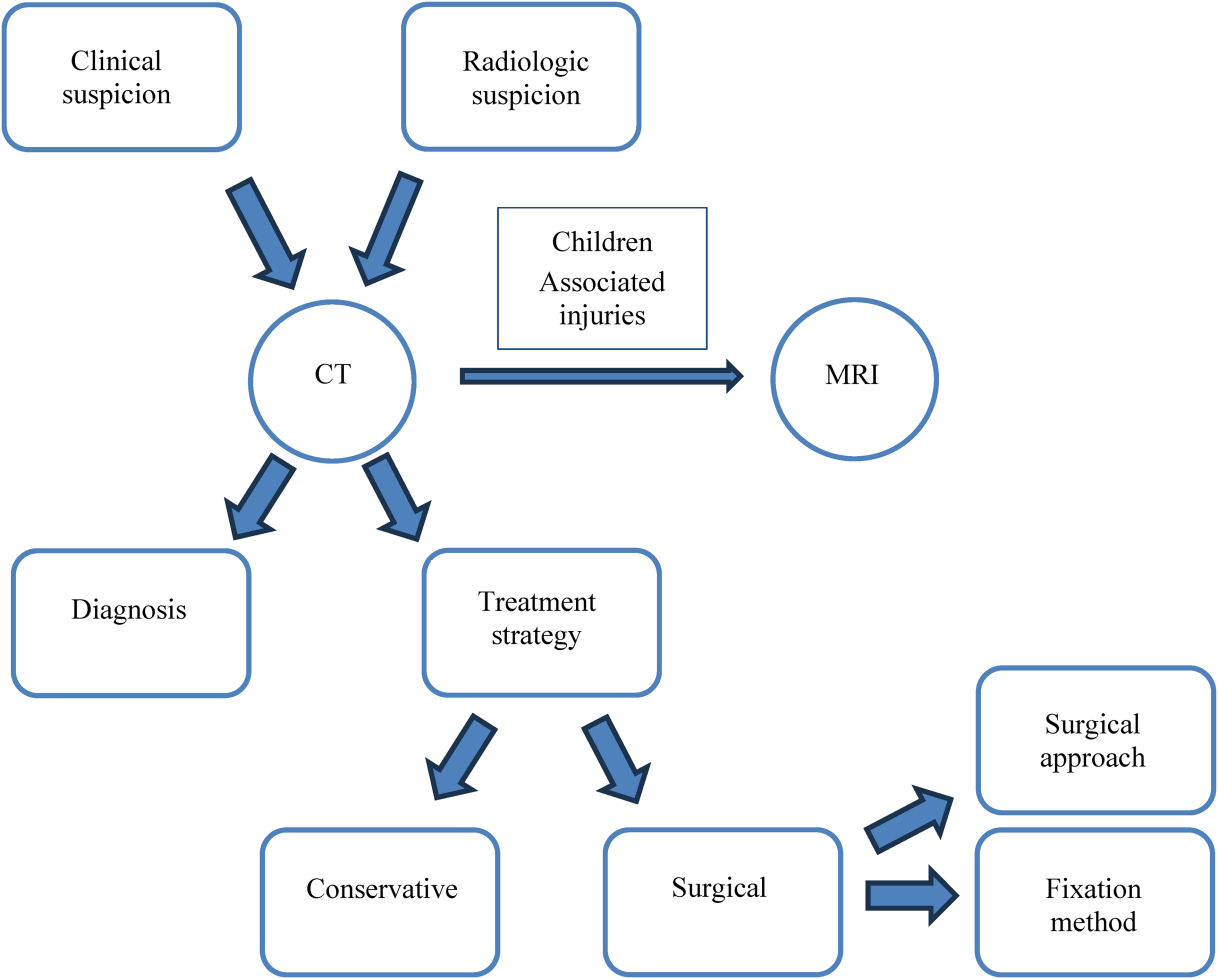
Diagnostic-therapeutic work-up.

Our case report showed the story of two adolescents with a capitellar shear fracture Type 1 and 4 according to Bryan-Morrey classification, surgically treated with open reduction and internal fixation by headless screws and K-wires, respectively, with complete recovery of function and radiological union at follow-up. According to our experience and the review of the literature, the diagnostic and therapeutic work-up of the pediatric shear fractures of the capitellum starts with a clinical and radiologic suspicion, based on the double contour sign or indirect signs of elbow injury (joint effusion, soft tissue edema) on standard radiographs. In pre-adolescents and adolescents, the diagnostics should be pursued to the CT, which not only allows the confirmation of the diagnosis, but also the choice of the treatment strategy, conservative or surgical. The CT scan also provides relevant information as the site where the fragment is displaced, which will guide the choice of the surgical approach, and its size, useful in the planning of the fixation method (Figure 3). The CT scanner is more widely available compared to MRI, that should be used for children due to the higher cartilaginous component, or in pre-adolescents and adolescent to clear associated injuries (ligamentous lesions, for instance).

The narrative review is limited by the impossibility of drawing statistical conclusions due to the predominance of case reports in the pediatric literature. The predominance of case reports has justified the performance of a narrative review rather than a systematic one. The description of complications and sequelae could be affected by the follow-up time of individual studies, varying between 4.5 months to 8 years. The limitation of retrospective case reports or case series are mainly represented by the recall and selection bias as well as the missing data related to the retrospective collection. To overcome these limitations, future research should rather aim to produce prospective studies, testing clinical guidelines and collecting predetermined clinical-radiologic variables and outcome measures, in the setting of multicenter studies. These data could aid to clearly define the diagnostic strategy that avoids the delayed diagnosis and treatment of these fractures, and standardize their management.

The interest in pediatric capitellar shear fractures lies not only in their challenging diagnosis and treatment but also in the sequelae that can develop and their further management. Future research should prospectively study these pediatric fractures, not only to draw decisional trees but also to find out the predictors of outcome, especially for the development of severe sequelae such as radiocapitellar osteoarthritis and osteonecrosis of the capitellum.

## Data Availability

The original contributions presented in the study are included in the article/Supplementary Material, further inquiries can be directed to the corresponding author.
